# UVA-Photosensitivity of cis-Diamminedichloro-Platinum(II)-Modified DNA

**DOI:** 10.1155/MBD.1995.137

**Published:** 1995

**Authors:** Dominique Payet, Marc Leng

**Affiliations:** 1 Centre de Biophysique Moléculaire CNRS, 1A avenue dela Recherche Scientifique Orléans cedex 2 F-45071 France

## Abstract

*cis*-Diamminedichloroplatinum(II)-modified oligonucleotides contain-ing either a single intrastrand cross-link at the d(GpG) site or an interstrand cross-link at the d(GpC/GpC) site, were irradiated with 320 to 400-nm light. Upon irradiation, the adducts are photosensitive. Among the photo-induced reactions, the cleavage of the coordination bonds between platinum and the G residues in the interstrand cross-link is demonstrated.


### UVA-PHOTOSENSITIVITY OF cis-DIAMMINEDICHLORO-PLATINUM(II)-MODIFIED DNA

Dominique Payet and Marc Leng

Centre de Biophysique Moléculaire, CNRS, 1A, avenue de la Recherche Scientifique
F-45071 Orléans cedex 2, France

### ABSTRACT

*cis*-Diamminedichloroplatinum(II)-modified oligonucleotides contain-ing either a single intrastrand cross-link at the d(GpG) site or an interstrand cross-link at the d(GpC/GpC) site, were irradiated with 320 to 400-nm light. Upon irradiation, the adducts are photosensitive. Among the photo-induced reactions, the cleavage of the coordination bonds between platinum and the G residues in the interstrand cross-link is demonstrated.

### INTRODUCTION

Although *cis*-diamminedichloroplatinum(II) (*cis*-DDP) is one of the most widely used anticancer drugs, the mechanism of its antitumor activity is not yet fully understood. Numerous results suggest that the cytotoxic action of the drug is related to its ability to react with cellular DNA (1-3). The main lesions have been characterized as bifunctional adducts including intrastrand and interstrand cross-links. The two major lesions are intrastrand cross-links at the d(GpG) and d(ApG) sites (4-5). The interstrand cross-links, a minor portion (5-10%) of the total lesions, are formed between two guanines on opposite strands (4-5) at the d(GpC/GpC) sites (6-7).

DNA and to a less extent *cis*-DDP ($e_{302}$ = 128 $M^{-1}.cm^{-1}$) (8) absorb ultraviolet light. It is now well-established that various photomodifications are induced in DNA by ultraviolet light. Less works have been devoted to the photochemistry of *cis*-DDP (9). Irradiation of *cis*-DDP induces the photoaquation of chloride ligands (8). Upon photolysis, the acidified solution of *cis*-$[Pt(NH_3)_2(H_2O)_2]^{2+}$ complex isomerizes to the *trans* form and the non-acidified solution changes to dark yellow with a concomitant increase of the pH of the solution. The later result might suggest a decomposition of the complex with a release of ammonia (8). Photoisomerizations have been reported for the *cis*-$[Pt(pyridine)_2Cl_2]^{2+}$ complex (10) and for the *cis*-$[Pt(glycinato)_2]$ complex (11).

To avoid the possible light effects, the platination reactions are generally carried out in the dark. We wanted to know whether the light could interfere with the stability of the lesions in *cis*-DDP-modified DNA. In this paper, we report some results showing that even in the wavelength region 320 to 400 nm, both the intrastrand and the interstrand cross-links are photosensitive. Among the photo-induced reactions, the cleavage of the coordination bonds between platinum and the G residues in the interstrand cross-link is demonstrated.

### MATERIALS AND METHODS

Deoxyguanosine-5'-monophosphate (dGMP) was purchased from Sigma. The oligodeoxyribonucleotides synthesized on an Applied Biosystems solid phase synthesizer, were purified by ion exchange chromatography on a Pharmacia FPLC system (12). T4 polynucleotide kinase was purchased from Ozyme, the radioactive products from Amersham and *cis*-DDP from Johnson-Matthey.

**Platination.** The reaction between *cis*-DDP and the pyrimidine-rich oligonucleotide d(TTCTCTTCTGGTCTTCTCTC) was performed in 10 mM $NaClO_4$, 5 mM sodium acetate, pH 3.6 at 37°C during 24 hours (12). The concentrations of the reagents were $3.10^{-2}$ mM. The platinated oligonucleotide was purified by FPLC (12). The duplex d(TCTCCTCTCGCTCTCCTTCT). d(AGAAGGAGAGCGAGAGGAGA) containing a single *cis*-DDP interstrand cross-link at the d(GpC/GpC) site was prepared as described (13).

**Irradiation.** The oligonucleotides, at a concentration of 5 mM, in 50 mM $NaClO_4$, 5 mM Tris-HCl pH 7.5, were irradiated at room temperature and during 20 minutes. At 5 mn intervals, aliquots were withdrawn and analyzed by gel electrophoresis using a 24 % polyacrylamide denaturing (8 M urea) gel. The oligonucleotides were $^{32}$P-labeled at the 5'-end by using T4 polynucleotide kinase (14) either before irradiation and then the enzyme was removed by two phenol treatments or after irradiation. The results were unchanged.

**Apparatus.** Absorption spectra were scanned by a Kontron Uvikon 810 spectrophotometer. Irradiations were done with an Osram HBO 200-W mercury lamp equipped with MTO J324a and MTO H325a filters (320-400 nm irradiation band). The fluence rate was 193 $W.m^{-2}$. Quantitation of the gels was done on a Molecular Dynamics Phosphorimager using Image Quant software version 3.3 for data processing.

### RESULTS AND DISCUSSION

It is known that upon platination, the absorption spectrum of DNA is modified with a bathochromic shift of the band centered at 258 nm and an increase in the absorbance (15-16). We have followed the reaction between *cis*-DDP and dGMP or DNA by ultraviolet absorption. The results (not shown) relative to DNA confirmed those of the literature. The platination of dGMP induces large changes in the spectra (fig. 1). In the range 250-265 nm the band is red-shifted as in the case of the platinated DNA. By contrast, the absorbance decreases. A qualitative explanation is that in the platinated DNA, there are two effects; one is a decrease of the absorbance due to the binding of *cis*-DDP to the G residues and the other is an increase of the absorbance due to the unstacking of the base residues (1-3). At l > 280 nm, the platinated species absorb more than the unplatinated dGMP. In the inset of fig. 1, are drawn the spectra in the range 300-400 nm, of a solution of dGMP and *cis*-DDP immediately after addition of the two reagents and after 24 h of incubation at 37° C, respectively. The conclusion of these experiments is that after platination, DNA and dGMP absorb the light above 320 nm.

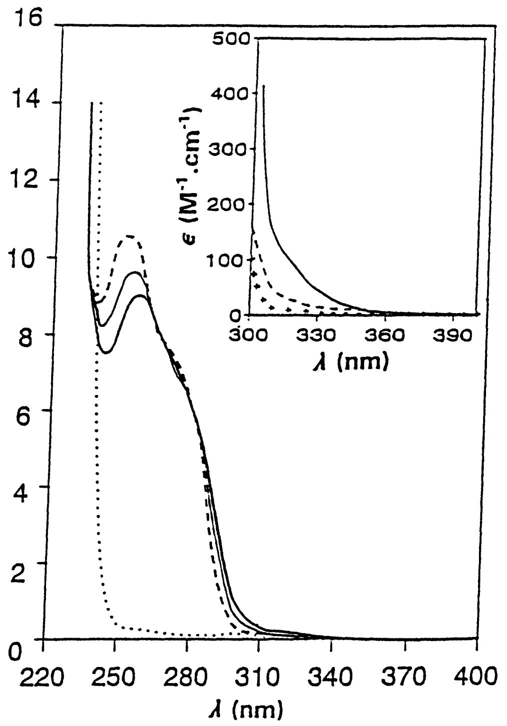

**Figure 1**. Absorption spectra of dGMP plus *cis*-DPP before incubation (- - -), after 8 h (——) and 24 h (— —) of incubation at 37° C. (....) corresponds to the solvent (10mM $NaClO_4$ plus 1% dimethylformamide).
The concentrations of dGMP and *cis*- DDP were 1 mM and 0.5 mM, respectively.
Insert: Absorption spectra of dGMP (+ + +), dGMP plus *cis*- DDP before incubation (- - -) and after 24 h of incubation (——) at 37° C.

To study the photoreactivity of *cis*-DDP-modified DNA, experiments were first carried out with the double stranded oligonucleotide d(TCTCCTCTC**GC**TCTCCTTCT).d(AGAAGGAGA**GC**GAGAGGAGA) containing an inter-strand cross-link at the single d(GpC/GpC) site. The platinated and the unplatinated duplexes were irradiated at room temperature. At 5 mn intervals, aliquots were withdrawn and analyzed by gel electrophoresis under denaturing conditions.

In the first set of experiments, only the pyrimidine-rich strand in the duplexes was $^{32}P$-labeled at the 5' end. Upon irradiation of the platinated duplex, several products are formed as revealed by the three new bands (b, c and d) on the gel (fig. 2, left). The bands (b) and (c) migrate much faster than the band (a) (the starting product) which means that the interstrand cross-link has been cleaved. The product (c) migrates as the unplatinated pyrimidine-rich strand (lane U) suggesting that the product (c) is indeed the pyrimidine-rich strand. To confirm this point, the oligonucleotide was eluted from the band (c), mixed with the purine-rich strand and then reacted with dimethylsulfate according to the procedure of Maxam and Gilbert (17). Analysis by gel electrophoresis (fig. 2, right) of the products of the reaction reveals the cleavage of the oligonucleotide at the level of the G residue in product (c) (this G residue in the platinated duplex does not react with dimethyl sulfate, fig. 2, right, lane +Pt). These results demonstrate that the N7 position of the G residue in the product (c) is no longer modified by *cis*-DDP and thus the light has induced the cleavage of the bond between Pt and the G residue in the pyrimidine-rich strand.

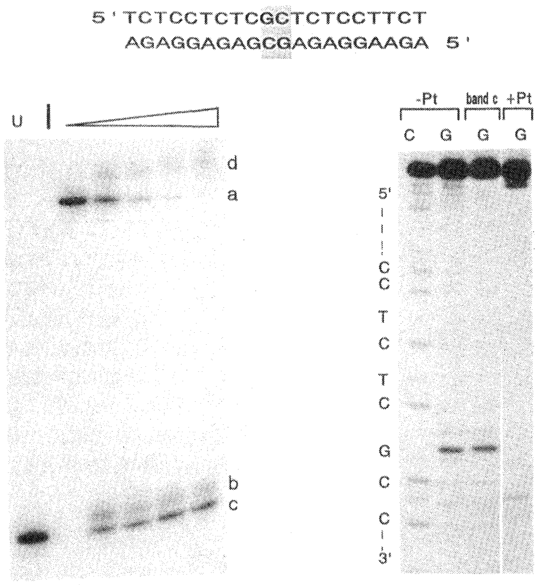

**Figure 2.** Autoradiogram of a 24% polyacrylamide denaturing gel of the duplex d(TCTCCTCTC**GC**TCTCCTTCT).d(AGAAGGAGA**GC**GAGAGGAGA) containing a *cis*-DDP-interstrand cross-link at the single d(GpC/GpC) site. Only the pyrimidine-rich strand in the duplex was $^{32}P$-labeled at the 5' end. Left. The lane (U) corresponds to the unplatinated pyrimidine-rich strand and the other lanes to the platinated duplex irradiated during 0, 5, 10, 15 and 20 mn. The band (a) corresponds to the starting product. Right. Reactivity of dimethylsulfate with the product (c) (band c, G) and with the platinated duplex kept at room temperature during 20 mn in the dark, (+ Pt, G). In the case of the platinated duplex (+ Pt, G), after the reaction with dimethylsulfate and prior to the piperidine step, the platinum residue was removed by treatment with NaCN (18-19). The lanes (- Pt, C and G) are relative to the Maxam-Gilbert-specific reactions for C and G (17) on the unplatinated pyrimidine-rich strand.

Product (b) is the pyrimidine-rich strand bearing an adduct since it migrates slower than the corresponding unplatinated strand. The product (b) was eluted from the gel. After incubation in 0.2 M NaCN (basic pH) to remove the bound Pt residue (18-19), it migrated as the unplatinated pyrimidine-rich strand and the G residue was reactive with dimethylsulfate. Although the nature of the adduct in the product (b) is unknown, these experiments show that upon irradiation of the interstrand cross-link, the bond between Pt and the G residue in the purine-rich strand is cleaved.

In the second set, all these experiments were repeated with the same platinated duplex in which only the purine-rich strand was $^{32}$P-labeled at the 5' end. The results (not shown) were similar to those presented in fig. 2. Thus, we conclude that the light has induced the cleavage of the bonds between Pt and the G residues in the purine- and pyrimidine-rich strands.

Other photo-induced reactions occur as revealed by the band (d) (in fact, a smear) in fig. 2. The products (d) migrate slower than the starting product showing that they contain interstrand cross-links differing in their chemical nature from that of the initial *cis*-DDP interstrand cross-link. These interstrand cross-links could result from a *cis-trans* isomerization (*trans*-diamminedichloroplatinum (II) cross-links G and C residues on opposite strands, (20)).

Several control experiments have been done. Under the same experimental conditons, the unplatinated duplex was stable. The stability of the platinated duplex after 20 mn at room temperature and in the dark, was verified by the reaction with dimethylsulfate. As shown in fig. 2 (right, lane +Pt), there is no cleavage of the pyrimidine-rich strand. Finally, all the experiments of irradiation were repeated, the solution containing an excess of plasmid DNA (DNA/duplex $\sim$ 10). The results were unchanged.

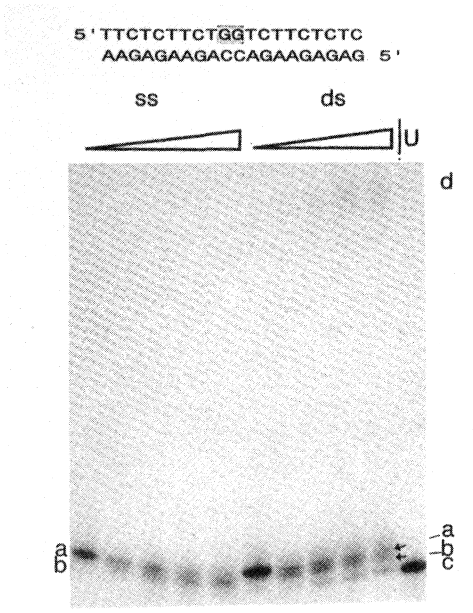

**Figure 3.** Autoradiogram of a 24% polyacrylamide denaturing gel of d(TTCTCTTCTGGTCTTCTCTC) containing a single intrastrand cross-link at the d(GpG) site. The platinated oligonucleotide $^{32}$P-labeled at the 5' end was irradiated alone (ss) or paired with its complementary strand (ds), and analyzed as described in the legend of the fig.2. The lane U corresponds to the unplatinated oligonucleotide. The band noted (a) is relative to the starting product.

The interstrand cross-link is highly photosensitive since after 20 mn of irradiation, it is almost completely destroyed. For comparison, the results relative to *cis*-DDP-modified oligonucleotides containing an intrastrand cross-link at the d(GpG) site are shown in fig. 3. The intrastrand cross-links are also photosensitive but to a less extent than the interstrand cross-link. In addition, the yields and the nature of the photo-products depend upon the conformation (single stranded versus double stranded) of the oligonucleotides. The product (c) was analyzed as described in the case of the interstrand cross-link. The results indicate that the product (c) is the unplatinated pyrimidine-rich strand and thus upon irradiation, the Pt-G bonds have been cleaved.

In conclusion, the intrastrand cross-link at the d(GpG) site and the interstrand cross-link at the d(GpC/GpC) site are photosensitive, even in the wavelength region 320-400 nm. Care should be taken to avoid to illuminate DNA or even cells, during and after the reaction of platination. Upon irradiation, several products are formed which implies several reactions. Among them, one corresponds to the cleavage of the coordination bonds between Pt and the G residues. Whether the adducts generated upon irradiation could react further with proteins bound to the platinated DNA is under study.

**ACKNOWLEDGMENTS**. We gratefully acknowledge Dr J. M. Malinge, A. R. Rahmouni and R. Dalbiès for discussions and comments. We are indebted to Dr M. Charlier for advice and help during the irradiation experiments. This work was supported in part by l'Association pour la Recherche contre le Cancer, la Ligue Contre Le Cancer and E. E. C. (project D1-92-002 and project CHRX-CT92-0016).

### REFERENCES

1- Eastman, A. (1987) *Pharmacol. Ther.* **34**, 155
2- Lippard, S. J. (1987) *Pure Appl. Chem.* **59**, 731
3- Reedijk, J. (1992) *Inorg. Chim. Acta* **198**, 873
4- Eastman, A. (1985) *Biochemistry* **24**, 5027
5- Fichtinger-Schepman, A. M. J.; Van der Veer, J. L., den Hartog, J. H. J., Lohman, P. H. M. & Reedijk, J. (1986) *Biochemistry* **28**, 7975
6- Lemaire, M.A., Schwartz, A., Rahmouni, A.R. & Leng, M. (1991) *Proc. Natl. Acad. Sci. U.S.A.* **88**, 1982
7- Hopkins, P. B.; Millard, J. T.; Woo, J.; Weidner, M. F.; Kirchner, J. J.; Sigurdsson, S. Th. & Raucher, S. (1991) *Tetrahedron* **47**, 2675
8- Perumareddi, J. R. & Adamson, A. W. (1968) *J. Phys. Chem.* **72**, 414
9- Maestri, M., Balzani, V., Deuschel-Cornioley, C. & von Zelewsky, A. (1992) in *Advances in Photochemistry* , eds. Volman, D., Hammond, G. & Douglas, N., John Wiley & Sons, **vol.17**, pp. 1-68
10- Moggi, L.; Varani, G.; Sabbatini, N. & Balzani, V. (1971) *Mol. Photochem.* **3**, 141
11- Scandola, F.; Traverso, O.; Balzani, V.; Zucchini, G. L. & Carassiti, V. (1967) *Inorg.Chim. Acta* **1**, 67
12- Marrot, L. & Leng, M. (1989) *Biochemistry* **28**, 1454
13- Payet, D.; Gaucheron, F.; Sip, M. & Leng, M. (1993) *Nucleic Acids Res.* **21**, 5846
14- Maniatis, T.; Fritsch, E. F. & Sambrook, J. (1982) *in Molecular Cloning: A laboratory Manual.* Cold Spring Harbor Lab., Cold Spring Harbor, New York
15- Macquet, J. P. & Butour J. L. (1978) *Biochimie* **60**, 901-914.
16- Munchausen, L. L. & Rahn, R. O. (1975) *Biochim. Biophy. Acta* **414**, 242
17- Maxam, A. M. & Gilbert, W. (1977) *Proc. Natl. Acad. Sci. USA* **74**, 560
18- Lippard, S. J., Ushay, H. M.,Merkel, C. M. & Poirier, M. C. (1983) *Biochemistry* **22**, 5765
19- Schwartz, A., Sip, M. & Leng, M. (1990) *J. Am. Chem. Soc.* **112**, 3673
20- Brabec, V. & Leng, M. (1993) *Proc. Natl. Acad. Sci. U.S.A.* **90**, 5345

**Received: January 20, 1995 - Accepted: February 16, 1995 - Received in revised camera-ready format: March 1, 1995**

